# A57 SUBOPTIMAL FIBRE CONSUMPTION AND ALTERED FIBRE METABOLISM IN CELIAC DISEASE THROUGHOUT THE GASTROINTESTINAL TRACT

**DOI:** 10.1093/jcag/gwae059.057

**Published:** 2025-02-10

**Authors:** M Wulczynski, M Constante, J Blom, G H Rueda, L Rossi, N El-Chaar, M G Surette, D Superdock, D Lawrence, M pinto-sanchez, D Armstrong, H Galipeau, E Verdu

**Affiliations:** Medical Science, McMaster University Faculty of Health Sciences, Hamilton, ON, Canada; Medical Science, McMaster University Faculty of Health Sciences, Hamilton, ON, Canada; Medical Science, McMaster University Faculty of Health Sciences, Hamilton, ON, Canada; Medical Science, McMaster University Faculty of Health Sciences, Hamilton, ON, Canada; Medical Science, McMaster University Faculty of Health Sciences, Hamilton, ON, Canada; Medical Science, McMaster University Faculty of Health Sciences, Hamilton, ON, Canada; Medical Science, McMaster University Faculty of Health Sciences, Hamilton, ON, Canada; Duke University School of Medicine, Durham, NC; Duke University School of Medicine, Durham, NC; Medical Science, McMaster University Faculty of Health Sciences, Hamilton, ON, Canada; Medical Science, McMaster University Faculty of Health Sciences, Hamilton, ON, Canada; Medical Science, McMaster University Faculty of Health Sciences, Hamilton, ON, Canada; Medical Science, McMaster University Faculty of Health Sciences, Hamilton, ON, Canada

## Abstract

**Background:**

Celiac disease (CeD) is a T-cell mediated enteropathy driven by gluten in people with risk genes HLA-DQ2 or DQ8. Many patients experience symptoms such as constipation and/or persistent mucosal inflammation despite adherence to a gluten-free diet (GFD) and are advised to increase dietary fibre. Preliminary results from gluten-immunized NOD-DQ8 mice revealed that supplemented inulin could improve mucosal healing. However, whether fibre is tolerated and metabolized in CeD is unknown.

**Aims:**

To investigate whether (1) fibre consumption in untreated and treated CeD compared to healthy volunteers (HV); (2) characterize microbial carbolytic capacity in the duodenum and feces.

**Methods:**

We conducted an analysis of CeD patients (newly diagnosed and GFD-treated) and HV attending our specialized McMaster adult CeD clinic. To investigate fibre consumption, participants completed a validated food frequency questionnaire (Victoria DQES v2), and the chloroplast *trnL-*P6 plastid marker was used to analyze the presence and diversity of plant DNA in feces at the time of sample collection (FoodSeq). To characterize fibre metabolism, fecal SCFA were measured using gas chromatography mass-spectrometry, and microbiota composition from duodenal aspirates was investigated by 16S rRNA gene Illumina sequencing of the V3V4 region. *In silico* predictions of metabolic functions and relative abundances of key enzymes were completed with PICRUSt2.

**Results:**

Most participants consumed fibre below the minimum recommendation (66% < 25g/day, Health Canada), despite diverse dietary plant consumption. Fibre did not correlate with self-reported gastrointestinal symptoms. Fecal SCFAs were lower in new CeD compared to HV, while GFD was partially rescued (CeD: 2799±927 vs HV: 5199±1616 μg/g, *p*<0.001; GFD: 4058±1283). Duodenal microbiota composition differed between all groups (Aitchinson Distance β-diversity, *p*<0.05). Abundance of enzymes related to fibre degradation (α-amylase K07405, fructan β-fructosidase K03332) were decreased in both CeD and GFD groups compared to HV (*p*<0.001), as was the abundance of several members of the *Prevotellaceae* family that had the greatest predicted contribution to this function (*p*<0.05).

**Conclusions:**

Newly diagnosed CeD patients have inadequate fibre consumption and lower microbial capacity to metabolize fibre in the lower and upper GI tract. Despite patients on GFD having the lowest fibre intake, microbial carbolytic activity was improved. Taken together with unpublished findings of inulin accelerating mucosal healing in mouse models of gluten sensitivity, the data should encourage clinical studies to define the effects of fibre supplementation on symptom management and mucosal healing in CeD during GFD.

**Funding Agencies:**

CIHRCeliac Canada

